# HarmoAtt-IK: an adaptive multimodal feature fusion network for real-time neural inverse kinematics

**DOI:** 10.3389/fnbot.2026.1769924

**Published:** 2026-04-24

**Authors:** Xukun Liu, Fengjuan Xie, Yu Liu, Jiaqiu Song

**Affiliations:** Northwest Institute of Mechanical and Electrical Engineering, Xianyang, Shaanxi, China

**Keywords:** adaptive multimodal attention, humanoid robots, neural inverse kinematics, real-time inference, zero-collection training

## Abstract

**Introduction:**

Recent advances in neural networks have introduced a new paradigm for robotic inverse kinematics. However, existing methods remain limited by insufficient feature extraction and suboptimal integration of multi-source information, preventing them from achieving high accuracy, broad generalization, and real-time performance on robots with diverse and complex kinematic structures.

**Methods:**

In this work, we propose HarmoAtt-IK, an adaptive multimodal neural inverse kinematics approach designed for real-time inference and zero-collection training. Built upon the CycleIK framework, the proposed method introduces a novel adaptive multimodal attention fusion mechanism (HarmoAtt) that dynamically integrates the complementary strengths of spatial, channel, and cross-dimensional attention. It employs a temperature-adaptive Softmax function coupled with a compact weight-generation network to perform multidimensional extraction and adaptive enhancement of input features. We further introduce a composite loss function integrating an improved Smooth-L1 loss, a sign-invariant quaternion loss, and a Shannon entropy regularizer to enhance training stability and overall accuracy. Leveraging forward differential kinematics, our method enables rapid, cross-platform deployment by generating training data solely from URDF models, eliminating the need for costly physical data collection and manual annotation.

**Results:**

Experimental evaluations on five humanoid platforms exhibiting substantial kinematic diversity demonstrate that HarmoAtt-IK attains maximum reductions of 76.4% in terminal positional error and 55.1% in rotational error relative to the baseline, while consistently improving the model’s inference success rate across all tested platforms by up to 5.76 percentage points.

**Discussion:**

These results indicate that the proposed HarmoAtt-IK significantly outperforms baseline methods in both accuracy and reliability across diverse kinematic structures, highlighting the effectiveness of the adaptive multimodal attention mechanism and composite loss design. This further supports its potential for scalable, real-time deployment on a wide range of robotic platforms.

## 1 Introduction

At the heart of modern robotic systems lies motion planning, which enables advanced functionalities such as dexterous grasping and precise manipulation. Central to motion planning is the problem of inverse kinematics (IK), which addresses the mapping from task space objectives to corresponding joint-space configurations. The fidelity of this inverse kinematic solution directly governs the positioning accuracy, computational efficiency, and real-time performance of a robotic system.

Recently, neural networks have emerged as a powerful data-driven paradigm for addressing inverse kinematics problems, owing to their remarkable capability for nonlinear mapping and generalization. These methods directly learn the mapping from end-effector poses to joint configurations by modeling the end-to-end relationship, bypassing the need for precise analytical models and facilitating strong generalization and real-time performance (Ardizzone et al., 2018; Ames et al., 2022; Kramar et al., 2022; Calzada-Garcia et al., 2025; Habekost et al., 2024; Limoyo et al., 2023). Despite these advantages, prevailing neural IK architectures suffer from notable limitations. A key shortcoming is the lack of effective mechanisms to fuse heterogeneous input data—such as target poses and inherent robot parameters—which restricts feature representation power and ultimately hinders further gains in accuracy and robustness.

To address these limitations, we introduce HarmoAtt-IK, an adaptive, multimodal neural inverse kinematics architecture designed for cross-platform deployment across heterogeneous robotic manipulators. Building upon the CycleIK framework (Limoyo et al., 2023), HarmoAtt-IK introduces a novel adaptive multimodal attention fusion mechanism, HarmoAtt, which dynamically integrates the complementary strengths of spatial, channel, and cross-dimensional attention—including SimAM (Yang et al., 2021), SE (Hu et al., 2018), Triplet (Misra et al., 2021), and Non-local (Wang et al., 2018)—in a synergistic manner to capture richer and more discriminative feature representations. At its core, a compact weight-generation module, governed by a temperature-controlled Softmax function, dynamically adapts the feature fusion strategy to the specific kinematic configuration of the target robot. This design facilitates multi-dimensional feature extraction and enables the adaptive refinement of input representations.

Inference performance across heterogeneous platforms is further strengthened through an adaptive hyperparameter optimization framework based on Multi-Fidelity Bayesian Optimization (MF-BO). This framework jointly optimizes network hyperparameters in a configuration-aware manner, yielding globally effective parameter settings across diverse robot morphologies. The training objective incorporates an enhanced Smooth-L1 loss, the Smooth Minimum Quaternion Loss (SMQL), along with a Shannon-entropy regularizer, enabling precise quantification of positional and rotational errors, resolving quaternion sign ambiguity and mitigating weight-distribution collapse. Leveraging forward differential kinematics, the framework autonomously generates dense training data directly from robot URDF models, supporting cross-platform deployment without reliance on any physical robot data acquisition or manual annotation.

The effectiveness of the proposed architecture is evaluated through extensive experiments on five representative humanoid platforms: NICOL (Kerzel et al., 2023), NICO (Kerzel et al., 2017), Franka Emika Panda (Haddadin et al., 2022), NASA Valkyrie (Radford et al., 2015), and Fetch (Wise et al., 2016). The proposed method is benchmarked against two widely used analytical IK solvers from MoveIt (Coleman et al., 2014)—the genetic algorithm–based BioIK (Starke et al., 2017) and the Jacobian-based TRAC-IK (Beeson and Ames, 2015), respectively. In addition, comprehensive system-level ablation studies are performed to quantify the contributions of individual architectural components and the proposed multimodal fusion strategy. Experimental results demonstrate that, across five humanoid robotic platforms with substantial kinematic diversity, HarmoAtt-IK achieves a maximum reduction of up to 76.4% in terminal positional error and up to 55.1% in rotational error compared with the baseline model, while consistently improving the model inference success rate across all tested platforms by up to 5.76 percentage points.

## 2 Related work

The implementation of high-precision and responsive robotic manipulators for complex tasks relies fundamentally on inverse kinematics (IK). Conventional IK solvers are predominantly categorized into analytical methods and numerical optimization approaches. Analytical methods provide closed-form solutions characterized by high computational efficiency and solution uniqueness, but their applicability is typically restricted to non-redundant manipulators with specific kinematic structures, limiting their generalizability (Chou and Liu, 2024; Qin et al., 2023; Kim et al., 2015). In contrast, numerical optimization approaches, such as Jacobian-based iterative algorithms and evolutionary strategies, facilitate motion planning for kinematically redundant systems. However, their iterative nature, which often requires the evaluation of a vast—or even infinite—solution space, incurs substantial computational cost, compromises real-time performance, and elevates the risk of convergence to local minima (Hernandez-Barragan et al., 2024; Luo et al., 2022; Carvalho, 2024).

The rapid advancement of deep learning has established neural network–based inverse kinematics (IK) solvers as a central focus of robotics research A widely adopted paradigm was introduced by Ardizzone et al. (2018), who employed invertible neural networks (INNs) to address the ambiguity inherent in inverse problems. However, their demonstration was limited to a three-degree-of-freedom (DoF) manipulator, leaving their applicability to high-DoF redundant systems largely unverified. Subsequent research has explored various architectures to address these limitations. IKFlow (Ames et al., 2022) extended the INN framework to redundant manipulators, achieving state-of-the-art precision. For high-DoF systems, Kramar et al. (2022) proposed a hybrid classification–regression architecture that partitions the joint space and iteratively refines predictions, thereby significantly enhancing accuracy. Other approaches prioritize efficiency: Calzada-Garcia et al. (2025) utilized deep neural networks (DNNs) to achieve robust generalization in redundant robots, while Habekost et al. (2024) implemented a multilayer perceptron (MLP) that provides deterministic, single-solution predictions with instantaneous inference and high accuracy. In contrast, the graph neural network (GNN)–based method developed by Limoyo et al. (2023), though accurate, typically incurs higher computational costs than INN- or MLP-based solvers.

While neural network-based methods hold considerable promise for solving inverse kinematics (IK) problems, several critical limitations persist in current approaches. A primary challenge lies in their architectural constraints. Predominantly reliant on fully connected or conventional convolutional layers, these models lack sophisticated mechanisms to effectively fuse heterogeneous data streams—such as target pose features and inherent robot configuration parameters—leading to suboptimal feature representation and extraction. Furthermore, their reliance on extensive, manually curated datasets for training inherently limits their ability to generalize to novel robotic morphologies and kinematic structures. Consequently, when applied to robots with diverse and complex topologies, existing solvers often fail to concurrently deliver the required triad of high precision, robust generalization, and real-time inference. Given the expanding landscape of robotic applications, there is a pressing need for an IK solver that is not only computationally efficient and real-time capable but also exhibits strong cross-platform adaptability and task-level generalization.

Attention mechanisms have garnered significant interest for their potential in robotic perception and control due to their ability to enhance feature representation. Notable advancements include parameter-free spatial attention (SimAM), which amplifies responses in critical regions without adding learnable parameters (Yang et al., 2021), and channel attention (SE) that recalibrates feature channels to emphasize salient information (Hu et al., 2018). To model broader contextual relationships, non-local attention mechanisms capture long-range dependencies across an input (Misra et al., 2021). More integrative approaches, such as triplet attention, facilitate cross-dimensional interactions between channel and spatial axes for a more holistic feature perception (Wang et al., 2018). Despite these innovations, individual attention modules are often insufficient to model the complex, high-dimensional, and multi-scale mappings required for robotic kinematics. Moreover, the naive aggregation of multiple such modules typically results in computationally prohibitive architectures. These challenges underscore the need for a lightweight and optimized attention-based architecture that can dynamically adapt to specific robot configurations and efficiently integrate multimodal features.

## 3 Method

### 3.1 Harmonized attention mechanism (HarmoAtt)

**Figure 1:**
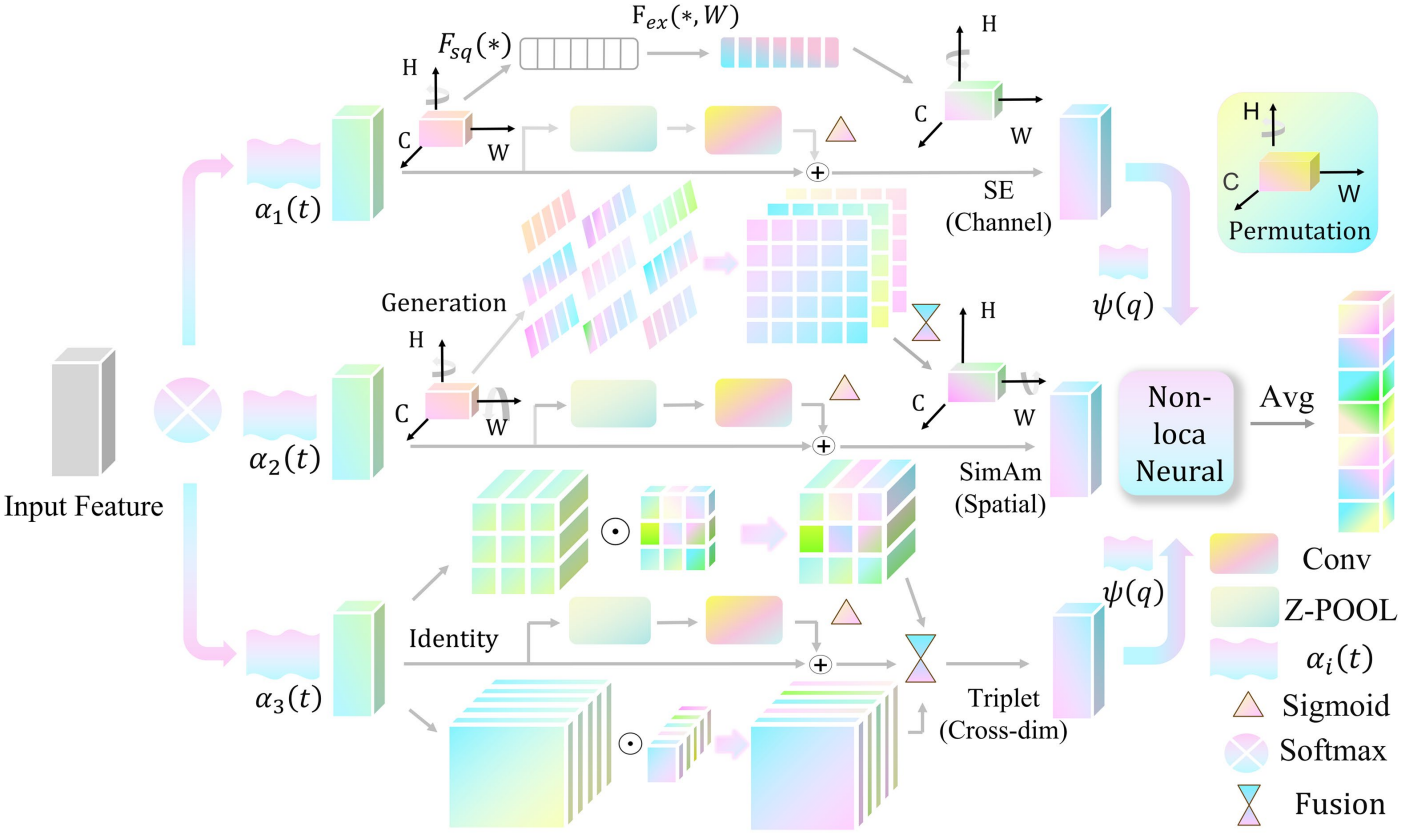
HarmoAtt: Adaptive multimodal fusion attention mechanism.

Figure 1 Existing neural inverse-kinematics methods suffer from limited discriminative feature extraction and inadequate integration of heterogeneous information. To address these limitations, we introduce HarmoAtt, a lightweight and adaptive multimodal attention mechanism that strengthens local feature representations while capturing long-range relational dependencies for comprehensive, dynamically adaptive feature refinement. Unlike naive aggregation, HarmoAtt employs a configuration-aware dynamic fusion strategy in which fusion weights are generated by a lightweight network conditioned on the robot’s Jacobian-based features, allowing the model to adaptively emphasize the most informative attention streams for the current kinematic state.

HarmoAtt performs localized feature enhancement via three parallel streams, targeting spatial distributions, channel-wise significance, and cross-dimensional interactions. To avoid parameter explosion from multi-branch operations, the outputs of all streams undergo channel dimensionality reduction and scale alignment via a shared linear projection prior to fusion.

Let the input feature tensor be $X\in {\mathbb{R}}^{B\times C\times L}$ , where B is the batch size, C the number of channels, and L the spatial dimension. We further employ the joint angle vector $q\epsilon {\mathbb{R}}^{n}$ from which X is generated via forward kinematics to compute configuration dependent kinematic features for adaptive fusion.

The three branches correspond to the following attention transformations Equation (1):


$$F_{i}=A_{i}(X)$$ (1)

Where $\;A_{1}$ , $\;A_{2}$ , and $\;A_{3}\;$ denote lightweight-optimized versions of the SimAM, SE, and Triplet modules, respectively.

To enable efficient fusion, we introduce a lightweight generation network that dynamically computes the fusion weights for each branch based on the robot’s current configuration features ψ(q) Equation (2):


$$\psi (q)=[\mathrm{DoF},\kappa (J(q)),r_{\text{null}}(q),∥J(q){∥}_{F}]$$ (2)

Here, κ(J) is the condition number of the Jacobian,reflecting manipulability; $r_{\text{null}}$ is the null-space dimension, indicating the degree of redundancy; $∥J(q){∥}_{F}$ is the Frobenius norm of the Jacobian, characterizing motion-coupling strength.

A configuration score is obtained via a trainable affine projection followed by normalization Equation (3):


$$s(q)=\text{Sigmoid}(W_{\mathrm{cfg}}\psi (q)+b_{\mathrm{cfg}})$$ (3)

Here, $W_{\mathrm{cfg}},b_{\mathrm{cfg}}$ are learnable weight matrix and bias term of the affine projection layer.

To capture multi-scale representations, we apply a five-scale feature pyramid network (FPN) to the input feature tensor X. The FPN uses dilated convolutions and residual connections to extract both fine-grained local details and long-range global dependencies.

The multi-scale convolution operator at scale is defined as Equation (5):


$${ℋ}_{s}(X)={\text{Conv}}_{k_{s},r_{s}}(X)+U_{s-1}({ℛ}_{s-1}(X))$$ (4)

Here, $k_{s}$ denotes the convolution kernel size, $r_{s}$ the dilation rate, and $R_{s-1}$ the cross-level residual mapping from the previous scale.

Features at each scale are independently normalized to eliminate scale discrepancies and maintain numerical stability Equation (5):


$${ℱ}_{s}=\text{LayerNorm}({ℋ}_{s}(X))=\gamma_{s}⊙\frac{{ℋ}_{s}(X)-\mu_{s}}{\sqrt{\sigma_{s}^{2}+\varepsilon }}+\beta_{s}$$ (5)

Here, $\mu_{s}=\frac{1}{d}\sum_{i=1}^{d}\;{\left[{ℋ}_{s}(X)\right]}_{i}$ and $\sigma_{s}=\sqrt{\frac{1}{d}\sum_{i=1}^{d}\;{\left[{ℋ}_{s}(X)\right]}_{i}-\mu_{s}){}^{2}}$ denote the mean and standard deviation of the feature vector ${ℋ}_{s}(X)$ at the s-th scale, respectively. $\gamma_{s},\beta_{s}\in {\mathbb{R}}^{d}$ are learnable affine parameters that modulate the normalized feature distribution, enabling stable feature.

Mapping and cross-scale alignment.

Fusion weights for features across scales are dynamically generated by a lightweight multi-layer perceptron (MLP) Equation (6):


$$z=W_{3}\text{GELU}(W_{2}\text{GELU}(W_{1}\text{Pool}(F_{s})+\rho_{1})+\rho_{2})+\rho_{3}$$ (6)

Here, $W_{1}\in {\mathbb{R}}^{c\times d_{1}}$ , $W_{2}\in {\mathbb{R}}^{d_{1}\times d_{2}}$ , $W_{3}\in {\mathbb{R}}^{d_{2}\times 5}$ are learnable parameter matrices, while $\rho_{1}\in {\mathbb{R}}^{d_{1}},\rho_{2}\in {\mathbb{R}}^{d_{2}}$ , $\rho_{3}\in {\mathbb{R}}^{5}$ are the corresponding bias vectors. GELU(⋅) denotes the smooth non-linear activation function, and the output $z\in {\mathbb{R}}^{5}$ represents the importance scores for the five-scale features.

Branch input mappings are obtained by fusing $z_{i}$ with the configuration score s(q) Equation (7):


$$z_{i}=W_{i}^{\left(p\right)}z+b_{i}^{\left(p\right)}+\vartheta_{i}s(q)\in {\mathbb{R}}^{h},i=1,2,3$$ (7)

Here, $\vartheta_{i}$ denotes the configuration-aware modulation coefficient, which controls the influence of the configuration score on the *i*-th branch.

For each branch feature zi∈Rh**z***i* ∈R*h*, we map it to a scalar weight score via global average pooling (GAP) and a nonlinear activation Equation (8):


$$u_{i}=\text{GELU}(w_{i}^{⊤}\mathrm{GAP}(z_{i}))$$ (8)

The fusion weights $\alpha_{i}$ are generated by a temperature-adaptive softmax function Equation (9):


$$\alpha_{i}=\frac{\exp(u_{i}/\tau )}{\sum_{j=1}^{3}\;\exp(u_{j}/\tau )},\tau \in {\mathbb{R}}^{+},i=1,2,3$$ (9)

Here, $z_{i}\;$ is a scalar representation obtained by applying global average pooling and nonlinear activation to the branch features. The temperature coefficient τ>0 is adaptively adjusted during training Equation (10):


$$\tau =1+\text{Sigmoid}(w_{\tau }^{⊤}\text{Pool}(X)+w_{s}s(q))\cdot \tau_{\max}$$ (10)

The final fused feature is computed as:


$$F_{\text{fused}}=W_{o}[F_{\mathrm{NL}}(\sum_{i=1}^{3}\alpha_{i}\text{Norm}(W_{s}F_{i}))]$$ (11)

Here, $W_{s}\in {\mathbb{R}}^{C\times (C/r)}$ is the shared channel compression matrix with reduction ratio r . Norm(·) indicates layer normalization, and $W_{o}$ is the output projection matrix. ${ℱ}_{\mathrm{NL}}(\cdot )$ represents the Non-Local mechanism, which captures long-range dependencies based on global similarity, enhancing the model’s ability to coordinate local and global interactions during training.

### 3.2 HarmoAtt-IK: integrated neural inverse kinematics framework

**Figure 2:**
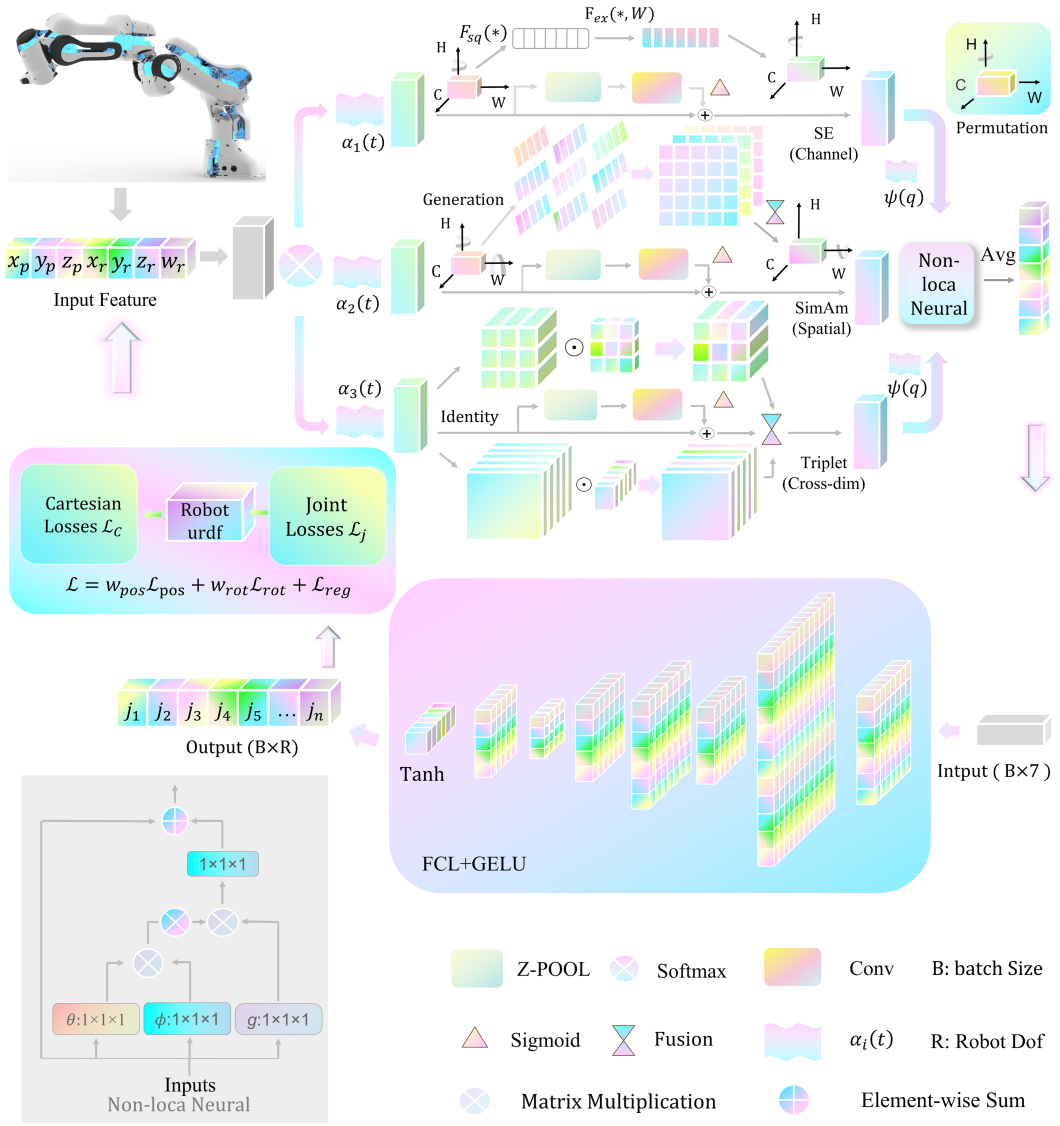
Neural inverse kinematics framework of HarmoAtt-IK.

HarmoAtt-IK constitutes an integrated neural inverse-kinematics (IK) framework (Figure 2) built upon a hierarchical modular architecture. By orchestrating multidimensional feature enhancement, adaptive attention fusion, neural IK reasoning, and joint-space mapping, the framework performs precise nonlinear transformations from Cartesian space to joint space, achieving high accuracy and robust generalization.

Here, 1 × 1 × 1 operation denotes the efficient channel-wise linear transformation employed for feature dimensionality reduction, projection, or channel attention computation. The functions θ(⋅) and ϕ(⋅) measure feature similarity across different positions, while g(⋅) performs feature transformation mapping. After Softmax normalization, the attention weights capture global positional dependencies, directly modeling long-range relationships and alleviating inherent limitations of convolutional operations.

The integrated multi-scale feature output $F_{\text{fused}}$ (Equation 11) is first transformed into a high-dimensional feature vector h via global average pooling and a non-linear projection layer Equation (12):


$$h=\text{ReLU}(W_{\text{proj}}\mathrm{GAP}(F_{\text{fused}})+b_{\text{proj}})$$ (12)

Here, GAP denotes global average pooling, and $W_{\text{proj}},b_{\text{proj}}$ are learnable projection parameters.

Subsequently, h is projected into joint space via a fully connected layer, yielding normalized joint angle predictions through a tanh activation Equation (13):


$$\hat{\theta }=\tanh(W_{\text{out}}h+b_{\text{out}})$$ (13)

Here, $W_{\text{out}},b_{\text{out}}$ represent the weights and bias of the output layer, respectively. The tanh(⋅) function serves as a saturating nonlinearity, restricting the predicted values within the range [−1, 1].

The predicted joint angles are obtained by denormalization Equation (14):


$$\theta_{\text{pred}}=\theta_{\min}+\frac{\hat{\theta }+1}{2}\cdot (\theta_{\max}-\theta_{\min})$$ (14)

The network loss function employs the improved tri-component structure, simultaneously constraining position error, orientation error, and an entropy-based regularization term for the attention distribution Equation (15).


$$ℒ=w_{\mathrm{pos}}{ℒ}_{\mathrm{pos}}+w_{\mathrm{rot}}{ℒ}_{\mathrm{rot}}+{ℒ}_{\mathrm{reg}}$$ (15)

Here, $w_{\mathrm{pos}}$ and $w_{\mathrm{rot}}$ denote the weighting coefficients for position and rotation errors, respectively, ${ℒ}_{\mathrm{reg}}$ represents the entropy-based regularization term.

To accurately evaluate the end-effector pose errors, an improved Smooth-L1 loss is applied Equation (16):


$${ℒ}_{\mathrm{pos}}=\frac{1}{B}\sum_{b=1}^{B}\;\sum_{k=1}^{3}\;l(p_{b}^{k},{\hat{p}}_{b}^{k})$$ (16)

Here, kϵ{x,,y,,z} represents the three spatial coordinates. $p_{b}^{k}$ is the target value of the *k*-th coordinate for the *b*-th sample (from the ground-truth training data), ${\hat{p}}_{b}^{k}$ is the corresponding inferred value predicted by the network through the inverse kinematics model. β is the Smooth-L1 threshold parameter governing the transition between the quadratic and linear regions.

The function l(·,·) denotes the improved Smooth-L1 loss, which robustly evaluates the error for each coordinate component. Its mathematical expression is as follows Equation (17):


$$l(p,\hat{p})=\{\begin{array}{c} \frac{{\left(p-\hat{p}\right)}^{2}}{2\beta }\;\text{if}|p-\hat{p}|\leq \beta \\ |p-\hat{p}|-\frac{\beta }{2}\;\;\;\text{otherwise} \end{array}$$ (17)

The loss function integrates an adaptive gradient modulation mechanism. When the error magnitude is below the threshold β , the quadratic (L2) loss is applied, allowing smooth fine-tuning with linearly decaying gradients.when the error exceeds β , the loss switches to linear (L1), mitigating gradient explosion under large errors and enabling dynamic, self-adaptive gradient regulation. During training, each component of the end-effector position error is independently optimized, ensuring balanced accuracy across all dimensions and preventing dominance by any single axis.

To address the inherent sign ambiguity of unit quaternions q∈SO(3) , where q and −q represent identical rotations, HarmoAtt-IK employs the Smooth Minimum Quaternion Loss (SMQL) Equation (18).


$$L_{\mathrm{rot}}=\frac{1}{N}\sum_{i=1}^{N}\;\min\{\sum_{j=1}^{4}\;l({\hat{q}}_{i,j},q_{i,j}),\sum_{j=1}^{4}\;l({\hat{q}}_{i,j},-q_{i,j})\}$$ (18)

Here, N denotes the batch size, $q_{i}$ and ${\hat{q}}_{i}$ represent the target and predicted quaternions, respectively. The function l(·,·) corresponds to the Smooth L1 loss, defined as follows Equation (19):


$$l(q,\hat{q})=\{\begin{array}{c} \frac{{\left(q-\hat{q}\right)}^{2}}{2\beta }\;\;\text{if}\;|q-\hat{q}|\;\leq \beta \\ |q-\hat{q}|-\frac{\beta }{2}\text{otherwise} \end{array}$$ (19)

The function l(·,·) provides smooth gradients for small angular deviations, facilitating precise end-effector pose adjustments, while transitioning to a linear loss for larger deviations to prevent gradient explosion and maintain numerical stability during optimization.

To mitigate attention weight collapse, a phenomenon wherein one or few branches dominate the fusion process, leading to representation capacity degradation and over-specialization, we introduce a Shannon entropy-based regularization term into the total loss function. This term encourages a more uniform distribution of attention weights across the three multimodal branches, thereby promoting feature diversity and enhancing model robustness Equation (20):


$$L_{\mathrm{reg}}=-\lambda \sum_{i=1}^{3}\alpha_{i}\log(\alpha_{i}+\varepsilon )\geq 0$$ (20)

Here, ε is a small constant for numerical stability, λ>0 is the balancing coefficient that controls the regularization strength. The regularization term attains its maximum when the attention weights $\alpha_{i}$ are uniformly distributed, promoting full utilization of multiple attention channels, and approaches zero as $\forall \alpha_{i}\to 1$ .

### 3.3 Platform-specific hyperparameter optimization

To enable the same network architecture to perform optimally across different robotic platforms without structural modifications, we employ a Multi-Fidelity Bayesian Optimization (MF-BO) framework to automatically determine the optimal set of hyperparameters for each target robot before training. This process ensures that the model is configured with platform-specific hyperparameters that maximize both accuracy and efficiency.

The hyperparameter vector for a given robot is defined as Equation (21):


$$\xi =\{\eta ,b,L,N_{i},w_{\mathrm{pos}},w_{\mathrm{rot}}\}\in \Xi$$ (21)

Here η denotes the learning rate, b the batch size, L the number of hidden layers, $N_{i}$ the number of neurons in the *i*-th layer, and $w_{\mathrm{pos}}$ and $w_{\mathrm{rot}}$ the loss weights for position and orientation, respectively.

For each target platform, we formulate the hyperparameter tuning as a constrained optimization problem Equation (22):


$$\min_{\xi \in \Xi }\;(J(\xi ;r),C(\xi ;r)),s.t.C(\xi ;r)\leq C_{\max}$$ (22)

Here, r represents the target robot platform identifier, J(ξ;r) denotes the validation error, quantifying the weighted loss on position and orientation predictions, C(ξ;r) represents the inference-time cost and structural complexity of the model.

For tractable optimization, these objectives are scalarized as Equation (23):


$$J_{\text{scalar}}(\xi ;r)=\lambda_{\mathrm{acc}}\frac{J(\xi ;r)}{J_{0}}+\lambda_{\mathrm{lat}}\frac{C(\xi ;r)}{C_{0}}$$ (23)

Here $J_{0},C_{0}$ are normalization coefficients, $\lambda_{\mathrm{acc}},\lambda_{\mathrm{lat}}$ denote the weighting factors for accuracy and latency, respectively.

A multi-fidelity Gaussian process model is constructed to approximate the objective function Equation (24):


$$y(\xi ,\ell )=f(\xi ,\ell )+\epsilon ,f(\xi ,\ell )\sim \mathrm{GP}(0,k_{\xi }(\xi ,\xi^{\prime })k_{\ell }(\ell ,\ell^{\prime }))$$ (24)

Here $k_{\xi }(\cdot )$ employs a Spectral Mixture Kernel to capture the non-stationary characteristics of the high-dimensional hyperparameter space, $k_{\ell }(\cdot )$ serves as the fidelity kernel.

We adopt a Cost-Aware Expected Improvement (CA-EI) acquisition function Equation (25):


$$\mathrm{Acq}(\xi ,\ell )=\frac{\mathrm{EI}(\xi ,\ell )}{c(\ell )},\mathrm{EI}(\xi ,\ell )=E[\max(0,y^{*}-y(\xi ,\ell ))]$$ (25)

Here $y^{*}$ denotes the best objective value observed so far, c(ℓ) represents the computational cost associated with fidelity level ℓ .

At each iteration, the next hyperparameter-fidelity pair is selected by Equation (26):


$$\left(\xi_{t+1},\ell_{t+1})=\arg\max_{(\xi ,\ell )\in \Xi \times ℬ}\mathrm{Acq}(\xi ,\ell \right)$$ (26)

Once candidate hyperparameters are identified, a hypergradient-based refinement step is applied for fine-tuning Equation (27):


$$\nabla_{\xi }L_{\mathrm{val}}(\varphi^{*}(\xi );\xi )=\frac{\partial L_{\mathrm{val}}}{\partial \xi }-\frac{\partial^{2}L_{\mathrm{val}}}{\partial \varphi \partial \varphi }{\left(\frac{\partial^{2}L_{\text{train}}}{\partial \varphi \partial \varphi }\right)}^{-1}\frac{\partial^{2}L_{\text{train}}}{\partial \varphi \partial \xi }$$ (27)

Local continuous optimization is achieved through $\xi \leftarrow \Pi_{\Xi }(\xi -\gamma_{\xi }\nabla_{\xi }L_{\mathrm{val}})$ , Where $\Pi_{\Xi }$ denotes the projection operator, $\gamma_{\xi }$ represents the hypergradient step size.

## 4 Experiments

We performed a comprehensive evaluation of HarmoAtt-IK across five humanoid robotic platforms with distinct kinematic structures: NICOL, NICO, NASA Valkyrie, Franka Emika Panda and Fetch. Model performance was assessed along three principal dimensions: convergence behavior, computational complexity analysis, and inference efficiency. In addition, system-level ablation studies on the NICOL platform rigorously examined the effectiveness of the HarmoAtt multimodal attention mechanism and quantified the contribution of each constituent module throughout training.

To ensure experimental fairness, all models were examined under identical conditions, including the same training, validation, test dataset splits, identical hardware configuration, and a fully consistent software environment. Pose samples in this test set were rigorously excluded from both the training and validation datasets, simulating the models’ performance in addressing scenarios involving previously unseen task commands.

### 4.1 Analysis of convergence performance comparison

**Figure 3:**
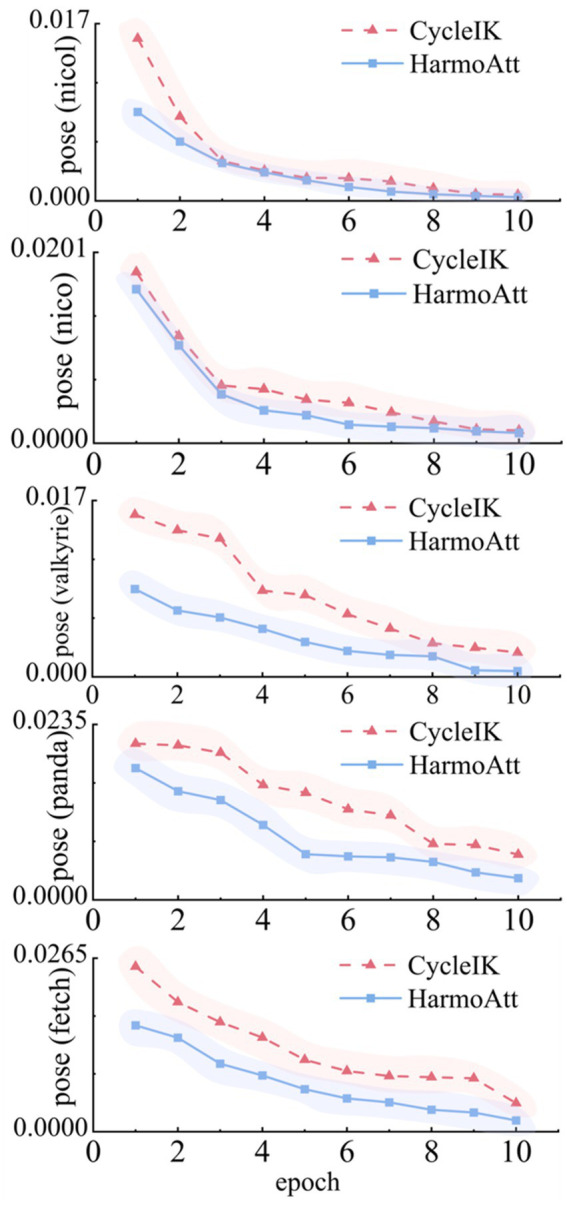
Pose-error convergence curves.

Figure 3 illustrates the pose error convergence curves of HarmoAtt-IK and the baseline method CycleIK across five robotic platforms with pronounced kinematic differences. All experiments were performed under identical datasets and training configurations, with the training schedule standardized to 10 epochs.

As illustrated, HarmoAtt-IK consistently outperforms the baseline in both convergence speed and stability across all platforms. On NICOL, the initial error decreases by approximately 45.2% compared with CycleIK, and the convergence curve remains notably smooth without pronounced oscillations. On NASA Valkyrie, the method yields more stable convergence and reduces the final position error by 76.4% relative to CycleIK. The baseline exhibits severe oscillations during epochs 4–6 and ultimately converges to 2.35 mm, which remains higher than the 2.12 mm achieved by HarmoAtt-IK at epoch 7. On the Franka Emika Panda platform, HarmoAtt-IK reaches the final accuracy level of CycleIK within only five epochs, demonstrating a clear advantage in training efficiency. On Fetch, the method reduces the early-stage error by more than 30% and consistently maintains a 40–65% improvement thereafter, resulting in a smoother convergence trajectory without significant oscillations.

**Figure 4:**
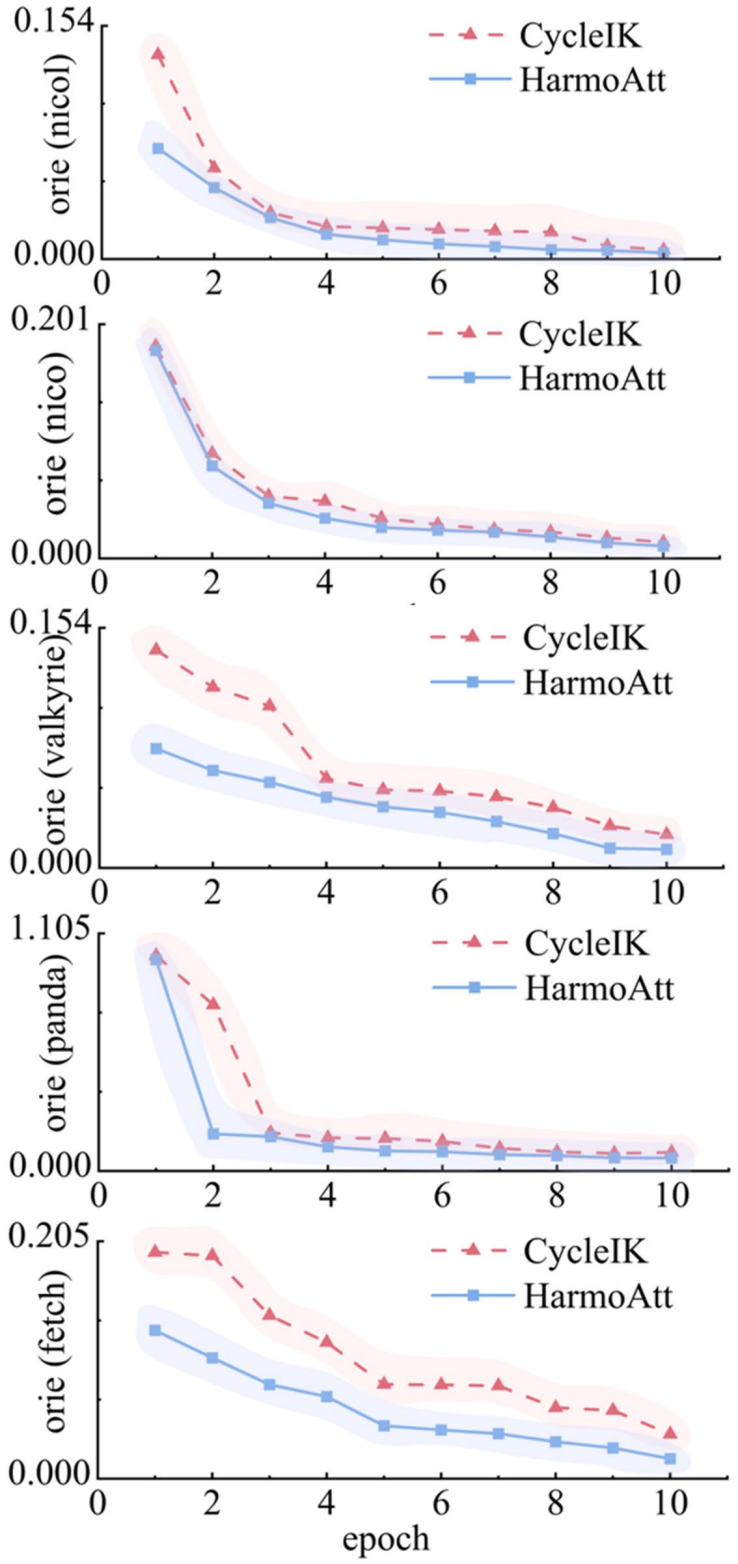
Rotation-error convergence curves.

Figure 4 illustrates the convergence curves of the rotational error. On the NICOL platform, HarmoAtt-IK reduces the initial rotational error by approximately 45.9% compared with CycleIK and exhibits a markedly smoother convergence trajectory, effectively suppressing the pronounced oscillations observed in CycleIK during epochs 4–8. On NASA Valkyrie, HarmoAtt-IK follows an approximately linear and monotonic convergence trend, decreasing the early-stage error by about 45.2% and reaching the final accuracy of CycleIK two epochs earlier. On the Fetch platform, HarmoAtt-IK consistently sustains lower errors throughout training, achieving a 55.1% reduction by the final epoch and effectively eliminating the severe convergence oscillations observed in CycleIK.

### 4.2 Computational complexity analysis

To quantify the efficiency of HarmoAtt-IK relative to the baseline CycleIK, we compare their parameter counts, floating-point operations (FLOPs) per inference, and memory footprints across all five robotic platforms. As shown in Table 1, HarmoAtt-IK introduces only a marginal increase in computational complexity while delivering accuracy improvements.

**Table 1:** Model complexity comparison.

Robot	Model	Parameters	FLOPs	Initial memory (MB)	Peak memory (MB)	Δ (MB)
NICOL	CycleIK	29.14 M	29.13 M	120.19	121.21	1.02
	HarmoAtt	31.82 M	31.80 M	130.53	131.55	1.02
NICO	CycleIK	11.51 M	11.50 M	52.46	53.48	1.02
	HarmoAtt	12.76 M	12.75 M	60.49	61.51	1.02
Panda	CycleIK	17.77 M	17.76 M	76.95	77.97	1.02
	HarmoAtt	18.99 M	18.98 M	84.72	85.74	1.02
Valkyrie	CycleIK	8.58 M	8.57 M	41.06	42.07	1.01
	HarmoAtt	9.90 M	9.89 M	47.80	48.82	1.02
Fetch	CycleIK	23.13 M	23.12 M	96.46	97.48	1.02
	HarmoAtt	25.54 M	25.52 M	104.50	105.53	1.03

“Initial Memory” denotes the storage occupied by model parameters; "Peak Memory” refers to the maximum GPU memory usage during inference; Δ represents the inference memory increments.

Across the five platforms, HarmoAtt-IK exhibits a parameter increase ranging from 7.2% (Fetch) to 15.4% (NICOL) relative to CycleIK. Similarly, the proposed method raise FLOPs by between 7.0% (Fetch) and 15.9% (NICOL), depending on the robot configuration. During inference, our measurements indicate that the inference-time GPU-memory increase for both HarmoAtt-IK and CycleIK is on the order of 1 MB (≈1.02 MB in our tests). This result, obtained under identical hardware and software conditions using PyTorch’s standard memory-profiling API, implies that the architectural modifications in HarmoAtt-IK do not significantly elevate the baseline activation memory required for solving inverse-kinematics tasks.

The observed increases in parameters and FLOPs translate to an absolute inference time increase of approximately 0.2–0.3 ms per sample. For typical robot control loops operating at 1 kHz (1 ms cycle time), this latency remains well within the control budget.

### 4.3 Multi-method performance benchmarking

Table 2 summarizes the inverse-kinematics performance of HarmoAtt-IK and the CycleIK baseline across five robot platforms with substantial kinematic differences. All experiments were conducted under identical hardware and testing conditions on a laptop equipped with an Intel Core i9 14900HX CPU and an NVIDIA GeForce RTX 4060 GPU, running Ubuntu 20.04 with PyTorch 2.4.1 and CUDA 12.1. The evaluation further included comparisons on the same test set with BioIK (genetic-algorithm–based) and Trac-IK (Jacobian-based).

**Table 2:** IK solving performance across robotic platforms.

Robot	Method	Poseᵃ (mm)	Roteᵃ (°)	Succ (%)	Inference time (ms)
NICOL	HarmoAtt-IK	1.82	0.48	98.29	0.68
	CycleIK	1.86	0.55	98.16	0.39
	BioIK	0.54	0.09	99.87	1.00
	TRAC-IK	6.62	1.10	98.49	1.00
NICO	HarmoAtt-IK	2.82	0.79	96.73	0.62
	CycleIK	3.16	0.93	96.07	0.36
	BioIK	24.1	5.72	92.10	1.00
	TRAC-IK	20.7	4.92	93.21	1.00
Valkyrie	HarmoAtt-IK	1.55	0.65	98.43	0.66
	CycleIK	2.89	1.14	96.03	0.39
	BioIK	2.10	0.43	99.41	1.00
	TRAC-IK	66.9	13.8	80.52	1.00
Panda	HarmoAtt-IK	4.81	2.31	90.99	0.42
	CycleIK	6.18	2.75	87.38	0.24
	BioIK	12.2	2.53	96.76	1.00
	TRAC-IK	44.0	9.56	87.46	1.00
Fetch	HarmoAtt-IK	3.61	0.94	94.56	0.45
	CycleIK	6.12	1.79	88.80	0.24
	BioIK	0.35	0.05	99.91	1.00
	TRAC-IK	13.4	2.09	97.19	1.00

${\text{Pose}}^{a}$ : mean positional error (mm), $\;{\text{rote}}^{a}$ : mean rotational error (°), succ (%): IK success rate under 10 mm position and 20° rotation thresholds (Kerzel et al., 2023; Kerzel et al., 2020),time: runtime per sample (ms). BioIK and TRAC-IK are included to provide a comprehensive performance spectrum. BioIK prioritizes high accuracy through iterative optimization at the cost of higher latency, while TRAC-IK offers a faster but less robust alternative. Neural methods like CycleIK and HarmoAtt-IK are evaluated for their ability to balance sub-millisecond latency with competitive accuracy.

Experimental results indicate that BioIK attains the highest end-effector pose accuracy on certain platforms (e.g., NICOL and Fetch), but suffers markedly increased position and orientation errors on the NICO platform, revealing strong cross-platform sensitivity and limited generalization robustness. Trac-IK consistently underperforms across all evaluated platforms, showing particularly large position and orientation errors on the Valkyrie and Panda robots. HarmoAtt decreases the mean positional and rotational errors while maintaining an sub-millisecond inference latency, and further improves CycleIK’s IK success rate on all platforms, with a maximum gain of 5.76 percentage points.

### 4.4 Systematic ablation analysis

To systematically evaluate the contributions of individual optimized attention mechanisms in HarmoAtt on overall performance, we performed systematic ablation experiments on the NICOL platform, with CycleIK serving as the baseline. Convergence behavior and solution accuracy were assessed by comparing the loss trajectories of HarmoAtt with those of individual lightweight attention mechanisms across 10 training epochs.

**Figure 5:**
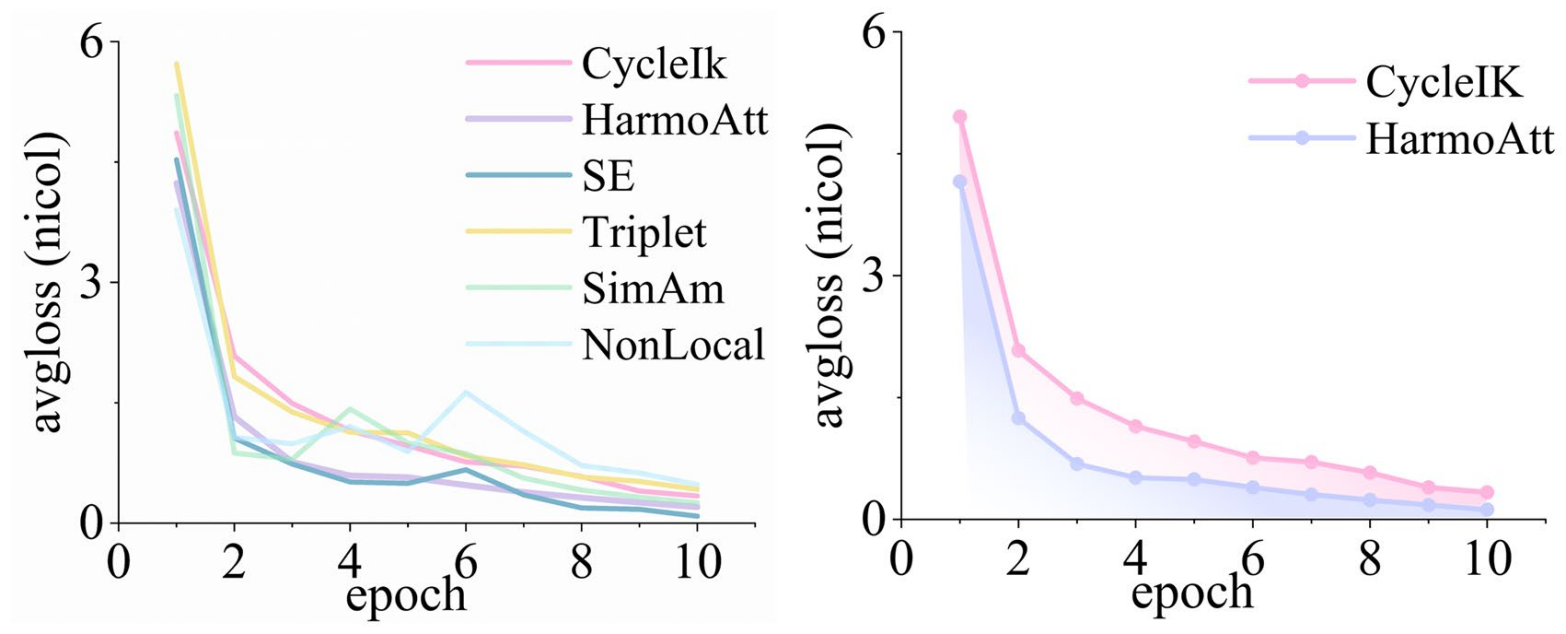
Loss convergence curves from component-wise ablation.

As illustrated, each individual attention mechanism exhibits distinct strengths and limitations during training. SE provides the largest convergence boost, even slightly outperforming full HarmoAtt-IK in final loss, yet suffers from strong oscillations and limited training stability. Triplet offers smooth, gradient-stable training but slower convergence, constrained by its local representational power. Non-local rapidly reduces loss initially, yet excessive global modeling introduces redundancy, leading to late-stage loss rebounds. SimAM minimizes loss early (epoch two) but over-reliance on feature saliency leads to overfitting and performance decay in later training. Each individual attention mechanism exhibits inherent limitations in addressing high-dimensional, nonlinear, and multi-scale robotic kinematic mappings, falling short of achieving an optimal balance among convergence rate, accuracy, and stability (Figure 5).

HarmoAtt integrates these optimized attention mechanisms, leveraging the synergy among multimodal features to fully exploit the complementary advantages of channel, local, global, and spatial attention. Across all training epochs, HarmoAtt reduces average per-epoch loss by approximately 38.7% compared with CycleIK, achieves its maximum improvement at epoch three (47.9% relative reduction) and reaches CycleIK’s final convergence level nearly three epochs earlier.

The necessity of the proposed MF-BO hyperparameter optimization framework is validated through an additional ablation study on the NICOL platform. Specifically, we compared two configurations of HarmoAtt-IK: one using the same hyperparameters as the CycleIK baseline (“w/o MF-BO”) and the other using platform-specific hyperparameters optimized by MF-BO (“w/MF-BO”). The results are summarized in Table 3.

**Table 3:** Ablation study on MF-BO hyperparameter optimization (NICOL platform).

Configuration	Position error (mm)	Rotation error (°)	Success rate (%)	Inference time (ms)
Baseline	1.86	0.55	98.16	0.39
HarmoAtt-IK (w/o MF-BO)	1.83	0.49	98.22	0.69
HarmoAtt-IK (w/MF-BO)	1.82	0.48	98.29	0.68

As shown in Table 3, HarmoAtt-IK with MF-BO achieves the lowest positional error and the highest success rate among the three configurations, confirming that MF-BO effectively tailors hyperparameters to the target platform and enables the HarmoAtt architecture to achieve optimal performance.

**Figure 6:**
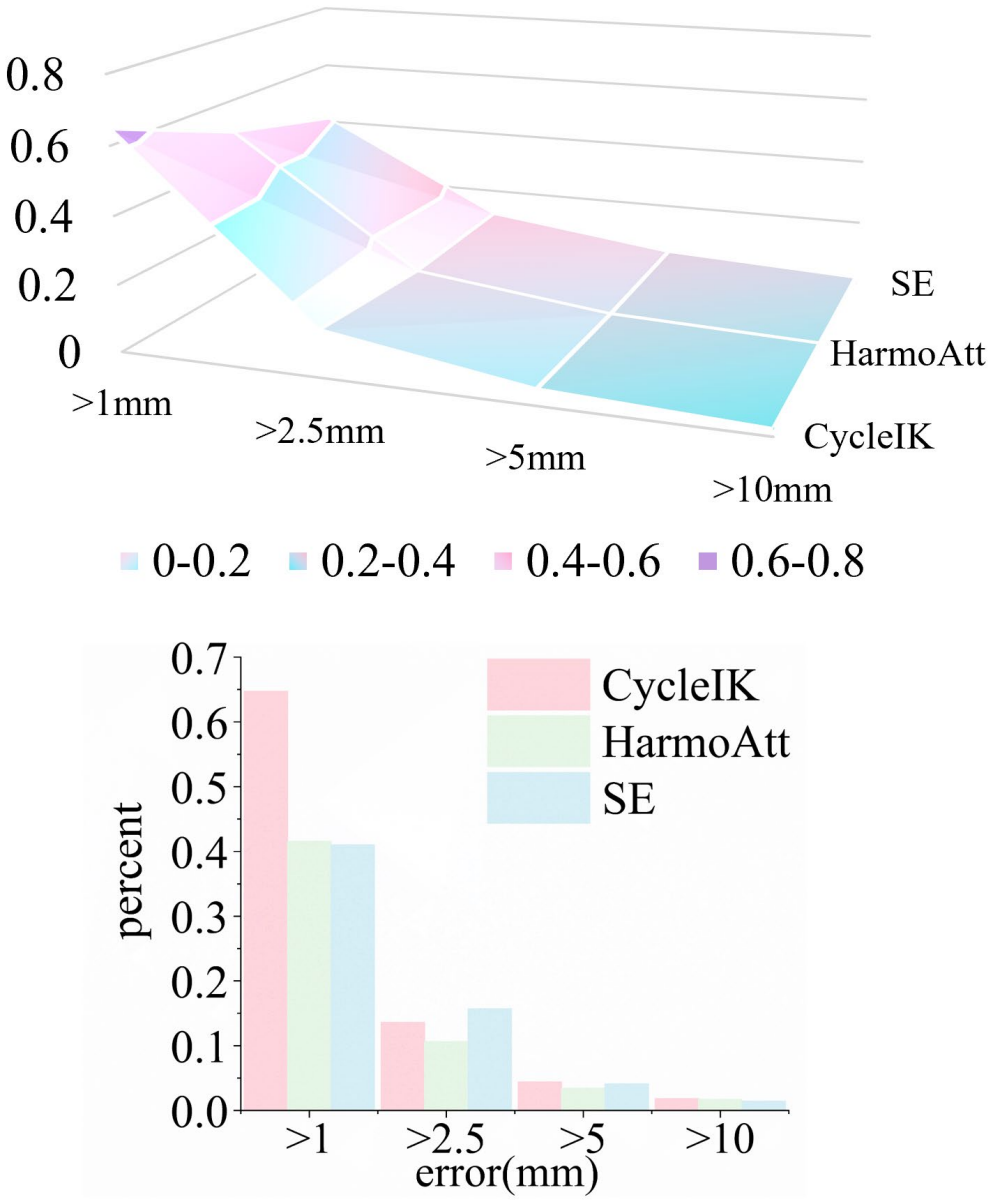
IK success rates across error thresholds for different methods.

We further compared the performance of the inverse kinematics (IK) solutions for HarmoAtt-IK, the baseline model and SE. The forward inference was conducted using the trained, converged model weights, with the results detailed in Figure 6.

Experimental results indicate that HarmoAtt-IK exhibits consistent and significant accuracy improvements across all threshold ranges. At the <1 mm threshold, HarmoAtt-IK improves the success rate by approximately 41.9% over CycleIK, performing only slightly below SE (by ~1%) while offering markedly more stable convergence. For the <5 mm threshold, the proposed method increases the success rate by about 18.1% relative to CycleIK and 9.7% relative to SE, substantially outperforming both the baseline and the individual mechanisms. These results validate the effectiveness of the proposed adaptive multimodal fusion strategy, demonstrating its ability to achieve an effective balance among high accuracy, generalization, and real-time IK performance.

## 5 Conclusion

In this paper, we present HarmoAtt-IK, an adaptive multimodal neural inverse kinematics framework that achieves robust, real-time performance across heterogeneous robotic platforms. Through extensive evaluations on five representative humanoid robots, HarmoAtt-IK demonstrated substantial improvements compared with baseline solvers, achieving up to 76.4% reduction in positional error and 55.1% reduction in rotational error, while inference success rates increased across all platforms, with the highest success rate improving from 88.8% on the Fetch robot to 94.6%.

The framework exhibits stable and efficient training dynamics, mitigating oscillatory behavior seen in the baseline model and single attention approaches. Ablation studies further confirm that the synergistic integration of lightweight attention modules—SimAM, SE, Triplet, and Non-local—is essential to effectively handle the high-dimensional, nonlinear nature of inverse kinematics. Compared with existing state-of-the-art methods, HarmoAtt-IK achieves superior accuracy while maintaining sub-millisecond inference latency. Beyond performance metrics, HarmoAtt-IK supports cross-platform deployment across diverse robotic morphologies without requiring any physical robot data acquisition or manual annotation.

Despite these advances, several avenues remain for future work. Reliance on simulation-generated data may not fully capture real-world physical complexities. Critical factors such as sensor noise, joint limits, and kinematic singularities are also not considered in the current evaluation. Addressing these aspects constitutes an important direction for future research.

## Data Availability

The original contributions presented in the study are included in the article/supplementary material, further inquiries can be directed to the corresponding author.
